# Invisible partners in care: Snapshot of well‐being among caregivers receiving comprehensive support from Veterans Affairs

**DOI:** 10.1002/hsr2.112

**Published:** 2019-01-31

**Authors:** Katherine E. M. Miller, Jennifer H. Lindquist, Maren K. Olsen, Valerie Smith, Corrine I. Voils, Eugene Z. Oddone, Nina R. Sperber, Megan Shepherd‐Banigan, G. Darryl Wieland, Jennifer Henius, Margaret Kabat, Courtney Harold Van Houtven

**Affiliations:** ^1^ Health Services Research and Development Service Durham VA Medical Center Durham North Carolina USA; ^2^ Department of Health Policy and Management University of North Carolina Chapel Hill North Carolina USA; ^3^ Department of Biostatistics and Bioinformatics Duke University Durham North Carolina USA; ^4^ Department of Population Health Sciences Duke University Durham North Carolina USA; ^5^ Division of General Internal Medicine, Department of Medicine Duke University Durham North Carolina USA; ^6^ Research Service William S. Middleton Veterans Memorial Hospital Madison Wisconsin USA; ^7^ Department of Surgery University of Wisconsin‐Madison Madison Wisconsin USA; ^8^ Caregiver Support Program Department of Veterans Affairs Washington DC USA

**Keywords:** caregiving, depression, informal carers, long‐term care, post‐9/11 Veterans, quality‐of‐life, Veterans

## Abstract

**Background and aims:**

Since May 2011, over 23 000 caregivers of Veterans seriously injured on or after September 11, 2001 have enrolled in the Program of Comprehensive Assistance for Family Caregivers (PCAFC). PCAFC provides caregivers training, a stipend, and access to health care. The aim of this study is to describe the characteristics of caregivers in PCAFC and examine associations between caregiver characteristics and caregiver well‐being outcomes.

**Methods:**

We sent a web survey invitation to 10 000 PCAFC caregivers enrolled as of September 2015. Using linear and logistic regressions, we examine associations between PCAFC caregiver characteristics and caregiver outcomes: perceived financial strain, depressive symptoms (Center for Epidemiologic Studies Depression Scale [CESD‐10]), perceived quality of Veteran's Veterans Health Administration (VHA) care, and self‐reported caregiver health.

**Results:**

We had complete survey data for 899 respondents. Since becoming a caregiver, approximately 50% of respondents reported reducing or stopping work. Mean time spent providing care was 3.8 years (median 3, IQR 1‐5) with an average of 4.9 weekdays (median 5, IQR 5‐5) and 1.9 weekend days (median 2, IQR 2‐2). The mean CESD‐10 score was 8.2 (median 7, 4‐12), at the cutoff for screening positive for depressive symptoms. A longer duration of caregiving was associated with having 0.08 increase in rating of financial strain (95% CI, 0.02‐0.14). Caregiver rating of the Veteran's health status as “fair” or better was a strong predictor of better caregiver outcomes, ie, self‐reported caregiver health. However, higher levels of education were associated with worse caregiver outcomes, ie, lower global satisfaction with VHA care, higher CESD‐10 score, and higher rating of financial strain.

**Conclusions:**

Higher depressive symptoms among longer duration caregivers, coupled with high rates of reductions in hours worked, suggest interventions are needed to address the long‐term emotional and financial needs of these caregivers of post‐9/11 Veterans and identify subpopulations at risk for worse outcomes.

## Abstract 2

The aim of this study is to describe the characteristics of caregivers in PCAFC and examine associations between caregiver characteristics and caregiver well‐being outcomes. We found higher depressive symptoms among longer duration caregivers, coupled with high rates of reductions in hours worked, which suggests interventions are needed to address the long‐term emotional and financial needs of these caregivers of post‐9/11 Veterans and identify subpopulations at risk for worse outcomes.

## 1 INTRODUCTION

In the United States, uncompensated family members and friends deliver the majority of long‐term services and support as informal caregivers. (Reaves & Musumeci^1^) An estimated 5.5 million informal caregivers, 2.3% of the adult population in the United States, provide medical care, emotional support, and/or physical assistance to Veterans (these caregivers are referred to as military caregivers henceforth).^2^ Approximately 1.1 million military caregivers provide care for Veterans who served^2^ post‐9/11. Post‐9/11 Veterans and their caregivers are younger than pre‐9/11 Veterans and their caregivers. Among post‐9/11 care recipients, 94.1% are aged 55 or younger. Estimates for post‐9/11 caregivers suggest 86.3% are 55 or younger and 37.1% are aged^2^ 18‐30. In contrast, among pre‐9/11 care recipients, 12.4% are aged 55 or younger. Estimates for pre‐9/11 caregivers suggest only 10.7% are aged^2^ 18‐30. Because of advances in medicine, Veterans who served post‐9/11 are surviving injuries with chronic disabilities, which Veterans from prior eras did not survive. Such disabilities may place greater demands on military caregivers. The demands due to chronic disabilities coupled with the younger population results in greater demands on caregivers for a longer period of time, as the caregivers and Veterans receiving care are younger.

Posttraumatic stress disorder (PTSD), traumatic brain injury (TBI), and depression are the signature injuries of the Operation Enduring Freedom/Operation Iraqi Freedom (OEF/OIF) conflicts.^3^ As a result of the signature injuries of OEF/OIF and the advances in medicine, the primary caregiving activities of post‐9/11 caregivers often differ from traditional caregiving activities. For example, post‐9/11 military caregivers provide assistance with fewer activities of daily living (eg, toileting) and instrumental activities of daily living (eg, managing finances) than civilian caregivers; however, they provide greater assistance helping “care recipients cope with stressful situations or avoid triggers of anxiety or antisocial behavior.”^2^ With differences in caregiving tasks coupled with an increased number of years providing care, the long‐term burden of caregiving on post‐9/11 military caregivers could include work strain, financial strain, and difficulty planning for the future (eg, caregiver's own retirement).^2^ Moreover, these younger caregivers may be balancing multiple demands, including young children and careers. Evidence of high levels of burden and strain for informal caregivers of patients with chronic diseases exists.^4^, ^5^, ^6^ However, the impacts of the expected prolonged informal caregiving by post‐9/11 military caregivers are currently unknown.

In addition to the differences in caregivers of pre‐9/11 Veterans to caregivers of post‐9/11 Veterans, caregivers of Veterans may have different informal caregiving experiences compared with caregivers of civilians. Prior work has found that “compared with national statistics on nonmilitary caregivers, military caregivers were found to be younger, serve as caregivers longer, and have greater caregiver burden, stress, and financial strain.” 2, 7 To further compound these challenges, caregivers of Veterans with PTSD, a signature injury in post‐9/11 Veterans, may have higher burden and more negative caregiving experiences compared with caregivers of Veterans without PTSD.^2^, ^8^ The RAND Hidden Heroes report identified that caregivers of post‐9/11 Veterans had approximately “five times the odds of meeting criteria for probable depression and scored an average of 19 points higher on anxiety symptoms” compared with noncaregivers. For context, caregivers of pre‐9/11 Veterans and nonmilitary caregivers had “twice the odds of meeting criteria for probable depression and scored between six and eight points higher on the anxiety scale.”^2^

Historically, National Caregiver Support Programs in the United States have been fragmented and limited in scope.^9^ Currently, the optimal combination of services and supports for long‐term caregivers to maintain or improve their health and well‐being is unknown. In May 2010, the United States Congress established the Program of Comprehensive Assistance for Family Caregivers (PCAFC) in title 1 of the Caregivers and Veterans Omnibus Health Services Act of 2010 (PL 111‐163). Caregivers of Veterans seriously injured in the line of duty on or after September 11, 2001 may be eligible for PCAFC.^10^ If eligible, PCAFC provides services and supports for the caregiver, including, but not limited to, a monthly stipend paid directly to the caregiver, access to health care if not already covered under a health insurance plan, education and training, travel, lodging and subsistence, respite care, and mental health services. The monthly stipend ranges from $600 to $2300. Stipends are assigned based on acuity of the Veteran and the amount of care provided by the caregiver, with tier 3 being the highest stipend and tier 1, the lowest. Tier amounts are calculated based on home health aide wages and adjusted for geographic variation. All enrolled caregivers completed standardized training through a contract with a nonprofit organization serving those with disabilities, Easter Seals. Other education programs include, but are not limited to, monthly educational calls (eg, “Choosing Your Words: Harnessing the Power of Communication”), peer support programs, and online self‐care classes. Through this legislation and resulting program, the VA Caregiver Support Program, the Veterans Health Administration (VHA), now provides family caregivers with an unprecedented level of support in the United States. Since its inception, over 35 000 caregivers have participated in PCAFC, and stipend payments have surpassed $1 billion through December, 2016, exceeding expectations of the program capacity and cost.^10^

The support provided to caregivers in PCAFC is unmatched in the United States. Gaps in the literature of caregiver health and well‐being of post‐9/11 caregivers exist. Additionally, the demographics and health and well‐being of caregivers enrolled in PCAFC are unknown. Because PCAFC provides unmatched support in the United States, understanding caregiver health and well‐being for those post‐9/11 caregivers receiving intensive caregiver supports and services in the United States is currently unaddressed in the caregiving literature. Examining PCAFC caregivers' health and well‐being outcomes, such as caregiver depressive symptoms and financial strain, contribute to the gaps in the existing literature on post‐9/11 caregivers and on caregivers receiving intensive supports and services in the United States. The primary objective of this study is to describe the characteristics of caregivers in PCAFC using survey data collected in September 2015. The secondary objective is to examine associations between caregiver characteristics and caregiver well‐being outcomes, including caregiver perceived financial strain, depressive symptoms, Veteran's VHA quality of care, and self‐reported health status. The findings from this study highlight those caregiver characteristics associated with more positive caregiver outcomes of well‐being in the study sample. While these findings are not generalizable to the entire population, they provide some insight regarding caregiver characteristics associated with increased risk for poor outcomes, which may help inform future research to develop appropriate interventions for these individuals.

## 2 DESIGN AND METHODS

### 2.1 Study sample

The analysis uses cross‐sectional data collected through a national survey of caregivers as part of a larger coordinated effort to survey caregivers of Veterans.^10^ For the analysis of the PCAFC participants, we determined a target sampling pool of 10 000 caregivers based on projected response rate of 50%. To ensure wide geographic representation of the target sample, we invited a 10‐percent stratified sample of caregivers applying for support from each Veterans Affairs Medical Center (VAMC) (n = 8118), and an additional nation‐wide group of caregivers who were surveyed 9 months earlier (n = 1882) to participate. We required all caregivers in the analysis to have been enrolled in PCAFC for at least 90 consecutive days as of September 1, 2015. We conducted the survey through a secure website hosted by a third‐party vendor, Intellica Corporation, from September to October 2015. Paper surveys were also available upon request. The final analytic cohort was n = 899 and included survey responses, which had no missing data in the outcomes modeled (described below). Because of the low response rate, the sample does not claim to be representative of the total population.

### 2.2 Measures

Caregiver health and well‐being outcomes hypothesized to be impacted by PCAFC include perceived financial strain, depressive symptoms, global rating of satisfaction with VHA care for the Veteran, self‐reported health, level of community engagement, positive aspects of caregiving experienced, and employment status since becoming a caregiver. These outcomes encompass metrics of well‐being potentially impacted by caregiver supports and services and outcomes that are commonly measured in caregiver populations, ie, geriatric care recipients to younger, disabled care recipients.^4^, ^5^, ^6^ We also measured caregiver and Veteran demographic characteristics and Veteran health status indicators. We examined all variables descriptively. Given the small sample size and generalizability limitations, we prioritized metrics of caregiver health and well‐being and covariates for regression modeling *a priori*. We identified four measures of greatest interest for regression modeling, as informed by the program office administering PCAFC: perceived financial strain, depressive symptoms, global rating of satisfaction with VHA care for the Veteran, and self‐reported health of the caregiver. The significance levels for analyses were *P* < 0.05.

### 2.3 Outcomes examined descriptively only

We examined three additional outcomes of interest: level of caregivers' community engagement, positive aspects of caregiving, and caregivers' current work status.

#### 2.3.1 Level of caregivers' community engagement

We measured the caregivers' level of community engagement as defined by the number of times the caregiver went out into the community in the prior month to shop, see a movie, attend a sporting event, volunteer, attend religious services, or do something else he/she enjoys.^11^ Response categories were “never,” “one time,” “two times,” “three times,” “four times,” or “five times or more.”

#### 2.3.2 Positive aspects of caregiving

We assessed positive aspects of caregiving as captured by Tarlow and colleagues' nine item, validated measure among caregivers to patients with dementia.^12^ Scores range from 9 to 45, where a higher score indicates more positive aspects of caregiving experienced. Respondents were asked to rate how much he/she agreed with nine statements referring to positive feelings due to caregiving as “disagree a lot,” “disagree a little,” “neither agree or disagree,” “agree a little,” “agree a lot.” For example, respondents are asked how much they agree with the statements such as “Providing help to the Veteran has made me feel strong & confident” and “Providing help to the Veteran has made me feel appreciated.” The positive aspects of caregiving has a Cronbach's *α* of 0.94 in our sample of caregivers.

#### 2.3.3 Caregiver's current work status

Caregivers were asked to select the option that best described their work status since becoming a caregiver (“I am working my usual hours for pay,” “I am working reduced hours for pay,” “I started working for pay,” “I started working more hours for pay,” “I stopped working for pay completely,” or “I was not working before and am not now.”)^11^, ^13^

### 2.4 Outcomes examined descriptively and with regression modeling

#### 2.4.1 Caregiver perceived financial strain

We measured perceived financial strain through the three‐item Impact on Finances subscale from the Caregiver Reaction Assessment. Responses included “strongly disagree,” “disagree,” “neither agree nor disagree,” “agree,” or “strongly agree” for statements regarding the degree of financial strain experienced. For example, caregivers were asked how much he/she agreed with the following statements: “It is difficult to pay for the things the Veteran needs”; “Caring for the Veteran puts a financial strain on me”; and, “My financial resources are adequate to pay for things that are required for caregiving”. Scores range from 3 to 15, where a higher score indicates higher strain. The caregiver perceived financial strain scale has a Cronbach's *α* of 0.73 in our sample of caregivers.^14^

#### 2.4.2 Caregiver depressive symptoms

We measured caregiver depressive symptoms through the Center for Epidemiologic Studies Depression 10‐item Scale (CESD‐10).^15^ Responses include “never,” “rarely,” “sometimes,” or “often” regarding statements of frequency of depressive symptoms experienced. For example, caregivers were asked how often “were you bothered by things that don't usually bother you” and “did you feel depressed.” The reference period is within the last week of survey administration. Scores range from 0 to 30, where higher scores indicate more depressive symptoms. Depending on the use of the CESD‐10, a score of more than or equal to 8 or more than or equal to 10 is often used to indicate screening positive for depressive symptoms and probable depression, respectively.^15^ The CESD‐10 has a Cronbach's *α* of 0.84 in our sample of caregivers.

#### 2.4.3 Caregivers' global satisfaction with VHA care for the Veterans

We measured caregivers' global rating of satisfaction with the Veterans' VHA care through a single item from the Consumer Assessment of Healthcare Providers and Systems (CAHPS) 2013 Health Plan survey.^16^ “Using any number from 0 to 10, where 0 is the worst health care possible and 10 is the best health care possible, what number would you use to rate all the health care the Veteran received at the VA?” Possible scores range from 0 to 10, where higher scores indicate better care.

#### 2.4.4 Caregivers' self‐reported health status

We measured caregivers' self‐reported health status through a single item used^13^, ^17^ in the SF‐36. Caregivers were asked “how would you say your health is now” with possible responses including “poor,” “fair,” “good,” “very good,” or “excellent.” This was dichotomized into (1) responses of “good,” “very good,” or “excellent” compared with (0) responses of “poor” or “fair” because of the distribution of responses across categories.

### 2.5 Key explanatory variables and covariates

The primary explanatory variables of interest were sociodemographics, including caregiver race (white versus all others), caregiver relationship to Veteran (spouse or significant other versus all others), caregiver's highest level of education (less than some college; some college, vocational/trade school or associate's degree; or, bachelor's degree or more), length of time as a caregiver, stipend tier (1—lowest, 2 or 3—highest), and Veteran health status (“poor,” “fair,” “good,” “very good,” or “excellent”). We also controlled for the following covariates: caregiver ethnicity (Hispanic or not Hispanic), Veteran status of the caregiver (Veteran or not Veteran), and caregiver health insurance status (private, public, military, or none). Regarding race, the question was a “select all that apply” where the possible responses included American Indian or Alaska Native; Asian; black or African American; Native Hawaiian or other pacific islander; white; other; or, prefer not to answer. If a respondent selected “other,” then he/she had the option of writing in his/her race. For regression modeling, race categories were collapsed to “white” and “all other” because of the distribution. Regarding Hispanic ethnicity, possible responses included yes, no, or prefer not to answer.

### 2.6 Statistical analyses

We calculated descriptive statistics for the sociodemographic characteristics of caregivers, caregiver well‐being outcomes, and sociodemographic characteristics of Veterans. Caregiver sociodemographic variables included age, race, marital status, sex, Hispanic ethnicity, Veteran status, length of time as a caregiver, average number of weekdays providing care, average number of weekend days providing care, relationship to Veteran, living distance from Veteran, highest level of education, insurance status, income, and tier level. We used multiple linear regressions to estimate the association between explanatory variables and each of these primary outcomes of interest: perceived financial strain, depressive symptoms, and global satisfaction with VHA care. The same explanatory variables were included simultaneously in all models; no variable selection was conducted. We used multivariable logistic regression to examine associations between the explanatory variables and caregiver's self‐reported health status (good/very good/excellent compared with poor/fair). Explanatory variables were checked for collinearity before being entered in the model; additionally, we examined model diagnostics (eg, residuals) for goodness of fit. All analyses were conducted with SAS v9.4 (SAS Institute, Inc, Cary, North Carolina).

### 2.7 Ethical considerations

VHA Handbook 1058_05 (VHA 2011) provides guidance about authorization of manuscripts that have been developed through nonresearch activities (ie, without institutional review board (IRB) approval under the authority of VHA operations). All VHA authors of this manuscript attest that the activities that resulted in producing this manuscript were not conducted as part of a research project, but as part of the nonresearch evaluation conducted under the authority of Caregiver Support Program. The status of this work as quality improvement and not research was also confirmed following review by the Research and Development Committee at the VA Durham Health Care System. Caregiver responses were kept confidential to the researchers and anonymous to the operational partners, the Caregiver Support Program.

## 3 RESULTS

### 3.1 Sample characteristics

The survey had a 14% response rate overall. Of caregivers who had complete survey data (n = 899), 88.0% were married; 66.3% identified as white; 78.5% identified as non‐Hispanic; 9.6% were also Veterans themselves; and 85.9% were the spouse of the Veteran receiving informal care (see Table 1). Mean caregiver age was 43.1, and 94.6% of caregivers were female. Moreover, 95.9% of caregivers lived in the same house as the Veteran receiving care, and 79.1% of caregivers had an education level higher than a high school diploma/General Education Development (GED). Over half of the caregivers indicated receiving health insurance through an employer or Tricare or both, while 17.6% of caregivers reported receiving Civilian Health and Medical Program of the Department of Veterans Affairs (CHAMP VA) health insurance through PCAFC participation. The mean length of time as a caregiver was 3.8 years. Finally, using Caregiver Application Tracker (CAT) data, we identified that 27.0% of respondents received tier 1 (the lowest stipend); 41.7% received tier 2; and 31.3% received tier 3 (the highest stipend).

**Table 1:** Caregiver demographics

	%	N = 899
Sex		
Female	94.6	(n = 850)
		
Age, years, mean (SD; median, IQR)	43.1 (11.9; 42.0, 33‐59)	(n = 844)
Marital Status		
Missing	0.1	(n = 1)
Married	88.0	(n = 791)
Living together, in a committed relationship	5.2	(n = 47)
Divorced/separated	3.5	(n = 31)
Widowed	0.8	(n = 7)
Single, never married	2.5	(n = 22)
Race		
American Indian or Alaska native	0.6	(n = 5)
Asian	3.6	(n = 32)
Black or African American	16.2	(n = 146)
Indicated multiple races	5.0	(n = 45)
Native Hawaiian or other Pacific islander	1.6	(n = 14)
Other	6.8	(n = 61)
White	66.3	(n = 596)
Ethnicity		
Hispanic or Latino	21.5	(n = 193)
Veteran Status		
Caregiver is a veteran	9.6	(n = 86)
Length of time as caregiver, in years, mean (SD; median, IQR)	3.8 (3.4; 3, 1‐5)	(n = 899)
Average days providing care per week days, mean (SD)	4.9 (0.4)	(n = 895)
Average days providing care per weekend days, mean (SD)	1.9 (0.3)	(n = 897)
Relationship to Veteran		
Spouse, significant other	85.9	(n = 772)
Parent	9.5	(n = 85)
Child	1.7	(n = 15)
Sibling	0.9	(n = 8)
Other	2.1	(n = 19)
Distance Relative to Veteran		
Missing	0.3	(n = 3)
In the same house	95.9	(n = 862)
Within walking distance	0.8	(n = 7)
Within 20‐minutes of driving distance from caregiver's home	2.2	(*n* = 20)
Between 20 minutes and an hour of driving distance from caregiver's home	0.6	(n = 5)
Over an hour of driving distance from caregiver's home	0.2	(n = 2)
Education Level		
Grade school/junior high	1.1	(*n* = 10)
Some high school	2.2	(n = 20)
High school graduate	15.5	(*n* = 139)
GED	2.1	(n = 19)
Trade/technical/vocational school	8.5	(n = 76)
Some college credit but no degree	25.9	(n = 233)
Associate's degree (AA or AS)	17.4	(n = 156)
Bachelor's degree (BA or BS)	17.5	(n = 157)
Post graduate work or graduate degree	9.9	(n = 89)
Caregiver Insurance Status^a^		
Missing	1.2	(n = 11)
Private insurance, through employer	20.1	(n = 181)
Private insurance, through private insurer	1.9	(n = 17)
Private insurance, through marketplace	2.9	(n = 26)
Medicare	4.8	(n = 43)
MediGap	0.8	(n = 7)
Medicare part D	1.2	(n = 11)
Medicaid	6.3	(n = 57)
Champ VA (not from CSP^b^)	8.0	(n = 72)
Champ VA (from VA Caregiver Support Program (CSP))	17.6	(n = 158)
Tricare	39.4	(n = 354)
VA	3.6	(n = 32)
Indian Health Service	1.0	(n = 9)
Other	5.0	(n = 45)
Have no insurance	2.7	(n = 24)
Individual Total Annual Income		
Missing	17.6	(n = 158)
Less than $10 000	29.5	(n = 265)
$10 000‐$19 999	11.8	(n = 106)
$20 000‐$29 999	11.5	(n = 103)
$30 000‐$39 999	9.1	(n = 82)
$40 000‐$49 999	6.1	(n = 55)
$50 000‐$59 999	6.0	(n = 54)
$60 000‐$79 999	5.4	(n = 48)
$80 000 or more	3.1	(n = 28)
Household Total Annual Income		
Missing	21.8	(n = 196)
Less than $10 000	6.1	(n = 55)
$10 000‐$19 999	9.3	(n = 84)
$20 000‐$29 999	10.5	(n = 94)
$30 000‐$39 999	12.4	(n = 111)
$40 000‐$49 999	10.4	(n = 93)
$50 000‐$59 999	9.6	(n = 86)
$60 000‐$79 999	12.6	(n = 113)
$80 000 or more	7.5	(n = 67)
Stipend Tier Level^c^		
1 (lowest)	27.0	(n = 243)
2	41.7	(n = 375)
3 (highest)	31.3	(n = 281)

a Categories are not mutually exclusive. Multiple responses were allowed.

b Caregiver Support Program.

c Stipend tier level as of July 2015 was determined through the Caregiver Application Tracker (CAT).

Table 2 displays the descriptive statistics for the outcomes of interest. Caregivers enrolled in PCAFC had a mean CESD‐10 score of 8.4 (median = 7.0, IQR 4‐6). A score more than or equal to 8 indicates screening positive for depressive symptoms.^15^ Caregivers enrolled in PCAFC had a mean score of perceived financial strain of 9.0 (median = 9.0; range 3‐15, where a higher score suggests higher perceived financial strain). Moreover, their satisfaction with the Veteran's VHA care was an average of 6.4 (median = 7.0, IQR 4‐9), where 10 is the best care possible. Additionally, 75.0% of caregivers enrolled in PCAFC reported a health status of “Good” or better, and 55.6% reported community engagement a maximum of two times in the month prior. Caregivers enrolled in PCAFC had a mean score of 35.1 (median = 36.0, IQR 29‐44) for positive aspects of caregiving (maximum score = 45). Additionally, only 36.9% of caregivers reported no change in employment status since becoming a caregiver, with 18.8% working usual hours for pay and 18.1% not working prior to or after becoming a caregiver; 54.4% of caregivers reported working less since becoming a caregiver, broken down as 16.7% working reduced hours for pay and 37.7% stopping work for pay completely. A total of 4.2% of caregivers reported starting to work more since becoming a caregiver, specifically with 3.1% beginning to work for pay and 1.1% increasing the hours worked for pay. Finally, those caregivers who indicated taking time off from work for caregiving (n = 284 or 80.0%) reported taking an average of 20.4 hours off in a typical month. Figures S1 to S5 present distribution of continuous variables.

**Table 2:** Descriptive statistics of outcomes

	Mean (SD; median, IQR)	**N**
Caregiver depressive symptoms (CESD‐10 score), mean (SD)	8.4 (5.9; 7, 4‐12)	(n = 899)
CESD‐10 score more than or equal to 8, screen positive for depression	48.1	(n = 432)
CESD‐10 score more than or equal to 10, probable depression	38.6	(n = 347)
Perceived financial strain, mean (SD)	9.0 (3.3; 9, 3‐15)	(n = 899)
Global rating of satisfaction with VA care, mean (SD)	6.4 (2.7; 7, 4‐9)	(n = 899)
Caregiver's Health Status, % (n)		
Caregiver's health status is poor or fair	25.0%	(n = 225)
Caregiver's health status is good or better	75.0%	(n = 674)
Number of Times of Community Engagement in Prior Month, % (n)		
Missing	0.1%	(n = 1)
Never	9.6%	(n = 86)
One time	21.8%	(n = 196)
Two times	24.3%	(n = 218)
Three times	17.1%	(n = 154)
Four times	11.7%	(n = 105)
Five or more times	15.5%	(n = 139)
Veteran VR‐12 Score		
Mental health score, mean (SD)	33.8 (10.9; 32.6, 26.2, 40.8)	(n = 855)
Physical health score, mean (SD)	32.1 (9.6; 30.7, 24.6‐37.3)	(n = 855)
Positive aspects of caregiving, mean (SD)	35.1 (8.3; 36, 29‐44)	(n = 893)
Current Work Status, % (n)		
Missing	4.5	(n = 40)
I am working my usual hours for pay	18.8	(n = 169)
I am working reduced hours for pay	16.7	(n = 150)
I started working for pay	3.1	(n = 28)
I started working more hours for pay	1.1	(n = 10)
I stopped working for pay completely	37.7	(n = 339)
I was not working before and am not now	18.1	(n = 163)
Hours taken off work per month, if working, mean (SD)	20.4 (17.8; 16, 8‐25)	(n = 284)

Abbreviations: CESD, Center for Epidemiologic Studies Depression Scale; VA, Veterans Affairs.

### 3.2 Associations between caregiver and veteran characteristics with caregiver well‐being

Regression model results are presented in Table 3. Having an education level of some college, vocational/trade school, or associate's degree compared with those with less than some college educational level was significantly associated with a 0.74 unit lower score of global satisfaction with VHA care (95% CI, −1.20 to −0.29; *P* = 0.001, *t* test); 1.14 unit higher CESD‐10 score (95% CI, 0.17‐2.11; *P* = 0.022, *t* test), and a 1.12 units higher rating of financial strain (95% CI, 0.57‐1.67; *P* < 0.001, *t* test). Having an education level of a bachelor's degree or higher compared with those with an educational level less than some college was significantly associated with a 2.08 unit higher CESD‐10 score (95% CI, 0.98‐3.18; *P* < 0.001, *t* test) and a 1.70 units higher rating of financial strain (95% CI, 1.07‐2.33; *P* < 0.001, *t* test). Additionally, a greater length of time as a caregiver was also significantly associated with each extra year having 0.19 higher CESD‐10 score (95% CI, 0.07‐0.30; *P* = 0.001, *t* test) and 0.08 increase in financial strain (95% CI, 0.02‐0.14; *P* = 0.013, *t* test). Caregiver rating of the Veteran's health status as “Fair” or better was associated with improved caregiver outcomes examined. Caregivers receiving the highest‐level stipend, tier 3 (compared with tier 1), had lower levels of financial strain and lower CESD‐10 scores.

**Table 3:** Association of selected caregiver characteristics and caregiver well‐being outcomes

	CESD‐10^a^^,^ b (*n* = 899)	Caregiver Perceived Financial Strain^c^^,^ d (*n* = 899)	Caregiver Global Satisfaction with VA Care^e^^,^ f (n = 899)	Caregiver Health Status is “Good” or Better^g^^,^ h (n = 899)
	Estimated Coefficient (Standard Error)^i^	Estimated Coefficient Standard Error)^i^	Estimated Coefficient (Standard Error)^i^	Odds Ratio (95% Confidence Interval)^j^
Caregiver white race	0.78 (0.41)	−0.50 (0.23) *P* = 0.030	−0.69 (0.19) *P* < 0.001	0.91 (0.66‐1.27)
Caregiver is spouse to veteran	1.17 (0.49) *P* = 0.018	−0.27 (0.28)	−0.08 (0.23)	0.88 (0.59‐1.33)
Caregiver's highest level of education is vocational/trade school or some college	1.14 (0.49) *P* = 0.022	1.12 (0.28) *P* < 0.001	−0.74 (0.23) *P* = 0.001	1.08 (0.72‐1.61)
Caregiver's highest level of education is Bachelor's degree or higher	2.08 (0.56) *P* < 0.001	1.70 (0.32) *P* < 0.001	−0.51 (0.26)	0.88 (0.56‐1.39)
Length of time spent as a caregiver	0.19 (0.06) *P* = 0.001	0.08 (0.03) *P* = 0.013	−0.03 (0.03)	0.97 (0.93‐1.01)
“Fair” or better veteran health status	−2.04 (0.47) *P* < 0.001	−0.91 (0.27) *P* < 0.001	0.98 (0.22) *P* < 0.001	1.82 (1.28‐2.60) *P* = 0.001
Tier level 2	−0.76 (0.47)	−0.27 (0.27)	0.01 (0.22)	1.57 (1.08‐2.29) *P* = 0.018
Tier level 3	−1.15 (0.50) *P* = 0.022	−0.65 (0.29) *P* = 0.022	0.33 (0.24)	1.37 (0.92‐2.03)

a Higher scores reflect more depressive symptoms.

b Linear Regression, R^2^ = 0.09; Adjusted R^2^ = 0.07.

c Higher scores reflect higher perceived financial strain.

d Linear Regression, R^2^ = 0.06; Adjusted R^2^ = 0.05.

e Higher scores reflect improved quality of care.

f Linear Regression, R^2^ = 0.07; Adjusted R^2^ = 0.06.

g The reference group included caregivers whose self‐reported health was “poor” or “fair”.

h Logistic regression, c‐statistic = 0.61.

i *P* values were estimated using *t* tests.

j *P* values were estimated using Wald Chi‐Squared test.

Linear regression models had adjusted R‐squared ranging from 0.05 to 0.07. The logistic regression had a c‐statistic of 0.61. The linear model fit diagnostics suggest the models explain a small percent of the variation around the mean. The logistic model fit diagnostic suggests the model's predictive accuracy is mediocre.

## 4 DISCUSSION

Informal caregiving is receiving increased national attention. The rapidly growing aging population as well as the number of seriously injured Veterans from OEF/OIF/Operation New Dawn (OND) create a growing need for family caregivers and greater attention to policies and programs, which support family caregivers in this role. In September 2016, the National Academies of Sciences, Engineering and Medicine Committee on Family Caregiving for Older Adults released a report outlining the current policies supporting family caregivers in the United States.^18^ The committee identified the inadequacy of current supports for caregivers and proposed a national strategy to increase screening and education for caregivers to mitigate the risks of caregiving. Additionally, the proposed strategy empowers health care providers to incorporate an evaluation of the needs and capacities of the patient's caregiver into the patient's management plan. The driving force behind these recommendations was to improve the quality of care provided for the patient/care recipient. The report highlights that understanding the caregiver experience is a critical piece to providing appropriate support that can enhance outcomes for both the caregiver and care recipient.

In an effort to understand further the caregiver experience of PCAFC caregivers, we examined established outcomes commonly reported in the caregiving literature. We found that PCAFC caregivers substantially decreased their labor force participation after becoming a caregiver. Overall, PCAFC caregivers also reported high levels of depressive symptoms. Our findings are consistent overall with the RAND Hidden Heroes post‐9/11 caregiver profiles. Increased depressive symptoms and perceived financial strain were also associated with longer caregiving duration. Caregivers with higher levels of education generally fared more poorly than those with lower levels of education, which bears further inquiry to understand why. It may be that higher educated caregivers expected to have more control over how they would spend their lives than lower educated caregivers. Thus, it may be possible that these higher educated caregivers fared more poorly and struggling more with the caregiving role, but this is merely speculation that bears more rigorous inquiry. Unsurprisingly, the receipt of a higher stipend was associated with decreased perceived financial strain. Finally, we found that the caregiver's report of the Veteran's health status was strongly associated with caregiver outcomes, with better Veteran health associated with better caregiver well‐being.

PCAFC caregivers experienced pronounced self‐reported changes in employment status since becoming a caregiver, with approximately half of caregivers reporting they were no longer working for pay or have reduced hours at work due to the demands of caregiving. Additionally, caregivers reported a very high average number of hours of work taken off due to caregiving. The reduction in labor force participation by younger caregivers is of concern, as reduced earnings in the present, and the potential for reduced earnings in the future will affect the amount of social security received in retirement. Eligibility for Medicare benefits could also be at risk, as 40 quarters of payments into the social security system is required. Moreover, these caregivers, if previously engaged with the workforce, may have been expecting to rely on 401‐K or other retirement plans acquired through employment when they reach retirement age.^19^ Decreased hours or leaving the work force can inhibit or even prevent younger caregivers from receiving employer contributions to their own retirement. This would also affect the ability to plan and save for their own future long‐term care. In response to the needs of caregivers, in June 2018, the VA Mission Act mandated financial training for PCAFC caregivers. As OEF/OIF/OND Veterans are surviving injuries, which previously would have been fatal, their typically younger spousal caregivers may be providing care for an extended period of years, compared with middle‐aged or older caregivers providing care to older adults. Understanding the impact of reduced work force participation and caregiving, over a prolonged period on post‐9/11 military caregivers labor force engagement and health and well‐being status, is critical for future research of long‐term comprehensive caregiver supports.

Providing care for a greater length of time was also associated with the caregiver having more depressive symptoms. A CESD‐10 score of more than or equal to 8 is often used to indicate a positive screening for depressive symptoms; PCAFC caregivers reported a mean CESD‐10 score of 8.4 (median 7, IQR 4‐12), suggesting a clinically significant level of depressive symptoms in this population. Moreover, an increased length of time as a caregiver was also associated with higher financial strain in PCAFC caregivers. Caregivers in PCAFC also face challenges providing care focused on the supervision and protection of Veterans with mental health and/or neurological disorders.^20^ Caregivers providing this type of care typically focus on providing emotional support, safety, assistance with the management of serious mental illnesses, and sleep disorders, among others, for the Veteran. Caregiver depressive symptoms and burden have been documented to be higher when caring for a Veteran compared with a civilian.^2^, ^7^ Given the higher levels of depressive symptoms at baseline exacerbated by the expectation of prolonged caregiving duration, there is considerable need for longitudinal research to understand the ramifications of intensive, long‐term caregiving on caregiver depressive symptoms, and financial well‐being.

Finally, we found that caregiver rating of the Veterans' health status as “Fair” or better was strongly associated with better caregiver well‐being outcomes examined, ie, fewer depressive symptoms, lower perceived financial strain, higher global rating of satisfaction with VHA care, and “Good” or better self‐reported health. This finding reinforces the need for interventions, which target the caregivers of Veterans who report that the Veterans' health status is “Poor/Fair.” While using a global measure of satisfaction does not allow us to differentiate across specialties, it provides useful perception of quality of VA care from caregivers of medically complex patients who may be coordinating the Veterans' health care across a variety of specialties. The lower satisfaction with the global quality of VA health care rating highlights warrants future research to identify what experiences are driving these lower satisfaction scores. To add context, the mean satisfaction score of 6.36 out of 10 observed in the responding caregivers is substantially lower than the mean satisfaction score of Veterans in the VHA overall, which was 8.6 according to calculations using the 2013 VHA Customer Satisfaction Report.^21^

This analysis has several limitations. First, the single‐group, cross‐sectional design of the analysis precludes reaching conclusions about causality. We do not have a control group to which we can compare well‐being outcomes among those not participating in PCAFC. Second, this analysis is subject to self‐selection bias of those who completed surveys compared with those who did not return the survey. The response rate was low, with only approximately 9% of caregivers included in the analysis. Self‐selection bias coupled with the low response rate limits generalizability of results and could under‐represent certain populations less likely to participate in the survey, eg, those with mental or physical health issues. In a project interviewing similar respondents, declined participation was often because of lack of time to participate due to caregiving demands. Thus, results could overestimate caregiver health and well‐being by missing the responses of more intensive caregivers. We were unable to perform nonrespondent analyses to determine whether the sample represents the larger population. In September 2015, PCAFC enrollees included 28.6% in tier 1 (the lowest stipend), 38.7% in tier 2, and 32.8% in tier 3 (the highest stipend). While these proportions roughly correspond with the descriptive statistics by tier in our cohort, we cannot compare respondents and nonrespondents by other metrics. Third, this analysis also does not consider other caregiving demands, such as childcare or caring for aging parents. Fourth, while we control for length of time a caregiver has been a caregiver, we are unable to control for length of time a caregiver has been in PCAFC because of data quality limitations. Fifth, the goodness of fit tests suggest the linear models explain a small percent of the variation around the mean and the logistic regression has mediocre predictive accuracy. Finally, we rely on self‐reported outcomes, including caregiver report of Veteran's health status. Future research should take into consideration how Veteran's health status is categorized, as caregiver‐reported Veteran health status may not correspond with the Veteran's health status documented in the medical record. A Veteran with many comorbidities documented in the medical record may not necessarily be categorized as being in “Poor” health by his/her caregiver.

Despite these limitations, these survey results are the first caregiver‐reported data on the participants in a new national program supporting caregivers comprehensively, PCAFC. These data complement, but are distinct from, the RAND “Hidden Heroes” report, because these caregivers are identified as receiving support and being caregivers of Veterans engaged in the VHA health care system.^2^ These findings contribute to the body of literature and operational understanding of the caregivers of Veterans who served post‐9/11. Griffin et al found slightly higher, but similar, results for employment changes for caregivers of Veterans with Traumatic Brain Injury (TBI) and polytrauma.^22^ These findings suggest that even with comprehensive caregiver support, there are individual factors that render the caregiver at risk for worse outcomes. Thus, these findings highlight the need for longitudinal studies to determine the impact of long‐term informal caregiving on caregiver health and well‐being. Moreover, these findings provide a unique description of caregivers of post‐9/11 Veterans participating in PCAFC, which is consistent with the overall RAND report profile of post‐9/11 military caregivers^2^ and of self‐reported data on PCAFC participants. As the type of caregiving in this population differs from the traditional expectations of caregiving, eg, not Activities of Daily Living (ADL) focused, it is critical for researchers to develop appropriate interventions to better serve these often younger caregivers of post‐9/11 Veterans, such as considering support services with options for pension support to help offset leaving the workforce. These findings of caregiver health and well‐being in a caregiver population receiving unprecedented level of systematic support in the United States illustrate the continued need to develop novel combinations of trainings and supports for caregivers to offset negative outcomes of caregiving challenging this population.

## FUNDING

This project is funded by the Department of Veterans Affairs, Caregiver Support Program, and Quality Enhancement Research Initiative (PEC 14‐272) and also received support from the Durham VA Center for Health Services Research in Primary Care and Geriatrics Research, Education and Clinical Center. This work was also supported by the Center of Innovation for Health Services Research in Primary Care (CIN 13‐410). Dr Voils is supported by a Research Career Scientist award from the VA Health Services Research & Development Service (RCS 14‐443). Megan Shepherd‐Banigan is supported by a VA OAA HSR&D PhD Fellowship TPP 21‐027. One of the funding bodies, the VA Caregiver Support Program, provided input to the study design, interpretation of data, and review of the manuscript prior to submission.

## CONFLICTS OF INTEREST

The authors declare that they have no conflicts of interest.

## AUTHOR CONTRIBUTIONS

Conceptualization: C. H. V. H., M. O., C. I. V., E. Z. O., G. D. W., C. H., M. C. K., J. H., M. K.

Data Curation: K. E. M. M., J. L, M. O., V. S.

Formal Analysis: K. E. M. M.

Funding Acquisition: C. H. V. H., M. C. K., J. H., M. K.

Investigation: K. E. M. M., J. L., M. O., V. S., M. S. B., C. H. V.H.

Methodology: J. L., M. O., V. S., N.S., C. H. V. H.

Project Administration: K. E. M. M.

Supervision: C. H. V. H., V. S.

Validation: J. L.

Writing—Original Draft Preparation: K. E. M. M.

Writing—Review and Editing: J. L., M. O., V. S., N. S., M. S. B., C. I. V., E. Z. O., G. D. W., C. H., M. C. K., J. H., M. K., C. H. V. H.

K. E. M. M., J. L., M. O., V. S. and C. H. V. H. had full access to all of the data in this study and take complete responsibility for the integrity of the data and the accuracy of the data analysis. All authors read and approved the final manuscript.

## Supporting information


**Figure S1.** Distribution of Caregiver Perceived Financial Strain
**Figure S2.** Distribution of Caregiver Score of Center for Epidemiologic Studies Depression Scale (CESD‐10)
**Figure S3.** Distribution of Caregiver Global Ratings of VA Healthcare
**Figure S4.** Distribution of Caregiver Zarit Subjective Burden Scores
**Figure S5.** Distribution of Caregiver Scores of Positive Aspects of Caregiving
