# Supplementary material for: Invisible partners in care: Snapshot of well‐being among caregivers receiving comprehensive support from Veterans Affairs
Source: Health Sci Rep. 2019 Jan 31;2(3):e112. doi: 10.1002/hsr2.112 (PMC6427058; doi:10.1002/hsr2.112)
Supplement: Supplementary file 1 — Figure S1. Distribution of Caregiver Perceived Financial Strain Figure S2. Distribution of Caregiver Score of Center for Epidemiologic Studies Depression Scale (CESD‐10) Figure S3. Distribution of Caregiver Global Ratings of VA Healthcare Figure S4. Distribution of Caregiver Zarit Subjective Burden Scores Figure S5. Distribution of Caregiver Scores of Positive Aspects of Caregiving [file HSR2-2-e112-s001.docx]

**Supplementary Materials**

**Supplementary Figure 1. Distribution of Caregiver Perceived Financial Strain**

We measured perceived financial strain through the three item Impact on Finances subscale from the Caregiver Reaction Assessment. Responses included “Strongly Disagree”, “Disagree”, “Neither Agree nor Disagree”, “Agree” or “Strongly Agree” for statements regarding the degree of financial strain experienced. Scores range from 3 to 15, where a higher score indicates higher strain.

**Supplementary Figure 2. Distribution of Caregiver Score of Center for Epidemiologic Studies Depression Scale (CESD-10)**

We measured caregiver depressive symptoms through the Center for Epidemiologic Studies Depression 10-item Scale (CESD-10). Responses include “Never”, “Rarely”, “Sometimes”, or “Often” regarding statements of frequency of depressive symptoms experienced. Scores range from 0 to 30, where higher scores indicate more depressive symptoms. Depending on the use of the CESD-10, a score of ≥8 or ≥10 is often used to indicate screening positive for depressive symptoms and probable depression, respectively.

**Supplementary Figure 3. Distribution of** **Caregiver Global Ratings of VA Healthcare**

We measured caregivers’ global rating of satisfaction with the Veterans’ VHA care through a single item from the Consumer Assessment of Healthcare Providers and Systems (CAHPS) 2013 Health Plan survey. Respondents were asked “Using any number from 0 to 10, where 0 is the worst health care possible and 10 is the best health care possible, what number would you use to rate all the health care the Veteran received at the VA?” Possible scores range from 0 to 10, where higher scores indicate better care.

**Supplementary Figure 4. Distribution of Caregiver Zarit Subjective Burden Scores**

We measured caregiver subjective burden using the Zarit Caregiver Burden instrument. The Zarit Caregiver Burden tool is a twelve-item scale indicating frequency of stress experienced. Respondents are asked to indicate “Never”, “Rarely”, “Sometimes”, “Quite Frequently”, or “Nearly Always”. It covers the elements often mentioned by caregivers as problems, including health, psychological well-being, finances, social life, and the relationship shared by the caregiver and care recipient. Scores range from 0 to 48, where higher scores indicate higher burden.

**Supplementary Figure 5. Distribution of Caregiver Scores of Positive Aspects of Caregiving**

We assessed positive aspects of caregiving as captured by Tarlow and colleagues’ nine item, validated measure among patients with dementia. Possible scores range from 9 to 45, where a higher score indicates more positive aspects of caregiving experienced. Respondents were asked to rate how much he/she agreed with nine statements referring to positive feelings due to caregiving as “Disagree a lot”, “Disagree a little”, “Neither Agree or Disagree”, “Agree a little”, “Agree a lot”.
